# Comparison between endoscopic and surgical treatment of screen-detected versus non-screen-detected colorectal cancers

**DOI:** 10.3332/ecancer.2009.142

**Published:** 2009-05-12

**Authors:** B Andreoni, C Crosta, A Sonzogni, ME Pirola, A Pavan, L Bisanti, C Senore, R Sassatelli, R Sguinzi, E Bertani, PP Bianchi, AC Chiappa

**Affiliations:** 1 Division of General-Laparoscopic Surgery, European Institute of Oncology, Milan 20141, Italy; 2 University of Milan, Milan, Italy; 3 Endoscopy Division, European Institute of Oncology, Milan 20141, Italy; 4 Pathology Division, European Institute of Oncology, Milan 20141, Italy; 5 Direzione Generale Sanita’ Lombardy Region; 6 Department of Epidemiology, Regional Health Authority, Milan, Italy; 7 G.I.S.Co.R. President

## Introduction

Since 2005, the Italian National Health System (NHS) has implemented a screening program for colorectal cancer for all citizens over 50. Screening tests are free for the target population (so-called Minimal Care Level guaranteed for all Italian citizens). Invitees are asked to take an immunological test for Faecal Occult Blood (FOBT) every two years. Individuals with a positive FOBT test are invited to undergo a total colonoscopy in an SSN-accredited Endoscopy Department.

Each Italian Region has a centre for the coordination of the screening programme, which employs a dedicated software that can trace the progress of each citizen within the programme:

- FOBT invitation (first round) compliance/non-compliance (reminder);
- FOBT positive cases: total colonoscopy, endoscopic or surgical treatment of screen-detected lesions plus follow-up;
- FOBT negative cases: FOBT invitation after two years (second round).

The ‘Screening Centre’ has a database that can provide a detailed, real-time status of the programme: it is therefore possible to compare the characteristics of screen-detected and non-screen-detected cancers.

The target population for the screening programme includes all citizens aged 50–70, except ‘high-risk’ subjects (family history; serious, persistent IBD; previous colorectal surgery; recent non-screen-related FOBT and/or colonoscopy; apparent digestive-tract symptoms (proctorrhagia, abdominal pain, bowel irregularities, etc)).

## Screen-detected lesions

Table 1 reports the results of the screening programme for 2006 and 2007, as registered at the Lombardy Screening Centre (9,500,000 inhabitants, 2,481,117 of whom make up the target population). Table 2 reports the results of the same screening programme in Milan (1,300,000 inhabitants, 323,976 of whom make up the target population). In both tables, detection-rate data for FOBT positive subjects who underwent total colonoscopy are shown in bold type: in 2006, in Lombardy, 721 carcinomas and 3369 ‘high-risk’ polyps were detected in asymptomatic patients, out of 12,293 total colonoscopies (PPV in colonoscopy after FOBT positive: 7.3 + 34 = 41.3%).

After completing the first round of the programme (all citizens of Milan were invited to undergo FOBT through a letter, followed by reminders to non-compliant subjects), in Milan, 327 carcinomas and 1370 ‘high-risk’ polyps were detected in asymptomatic patients out of 4907 total colonoscopies (PPV in colonoscopy after FOBT positive: 8 + 33.5 = 41.5%). Programme DR for carcinoma: 3.17%, Programme DR for ‘high risk’ polyps: 13.27%.

## Non-screen-detected lesions

Through the NHS archives, it was possible to analyse workload and results of colorectal-cancer treatment in clinical practice (‘symptomatic’ patients) over the same period of time as the NHS-activated screening.

## Screen-detected and non-screen-detected tumours treated at IEO (2006–8)

Table 3 shows colorectal-cancer cases treated at the European Institute of Oncology (a cancer centre in Milan that is one of the nine SSN-accredited endoscopy units for the screening programme, as well as a general surgery unit). Between January 2006 and August 2008, 503 operations for colorectal cancer were performed, 228 of which were carried out on patients, aged 50–69, from Lombardy. Of these 228 operations, 106 cases (45%) were screen detected and 38 of which (35.8%) for cancerous polyps. Over the same period, 122 operations were performed for non-screen-detected cancers (53% of the total), 14 of which (11.4%) for cancerous polyps.

Surgical radicalization (resection plus lymphadenectomy) after ‘complete’ endoscopic polypectomy was performed in 39 patients (27 screen detected and 12 non-screen detected).

## Discussion

Screen-detected tumours have a more favourable staging than non-screen-detected lesions, as demonstrated in Table 3, with a significantly lower incidence of pT_3–4_, pN+, M+ cases. Cancerous polyps are 35.8% of screen-detected carcinomas and only 11.4% of non-screen detected tumours.

During the first screening round (2006–7), colorectal-cancer incidence increased both in Lombardy and in Milan, as reported in the corresponding Tumour Registry. This increase is due to the diagnosis of colorectal cancer in asymptomatic subjects. Through tumour registries it will be possible to evaluate whether there is a future incidence decrease (which is probable, considering that during the screening ‘high-risk’ polyps are diagnosed and removed, that is definitely pre-cancer lesions).

Because of the screening programme, it is more often possible to detect lesions that were rare during the pre-screening era. In particular, a large number of ‘cancerous’ and ‘high-risk’ polyps are detected and treated. This has led to better knowledge of these lesions.

**Figure 1:**
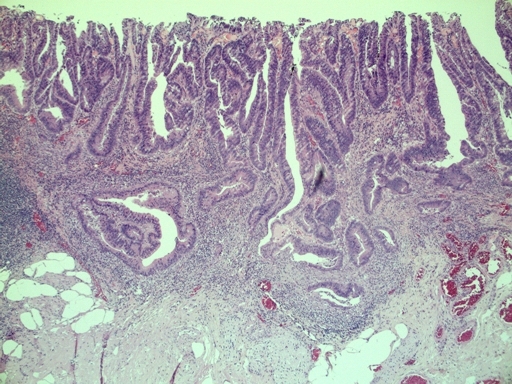
Pathological aspect of a malignant polyp.

**Figure 2:**
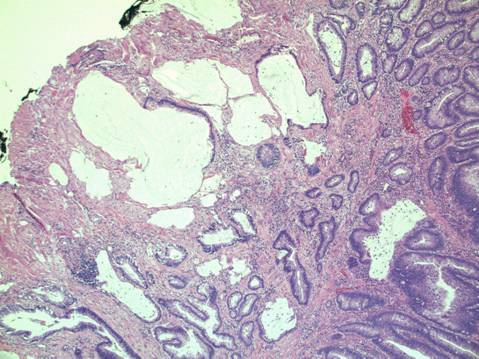
Histology—malignant polyp with mucinous aspects.

**Figure 3:**
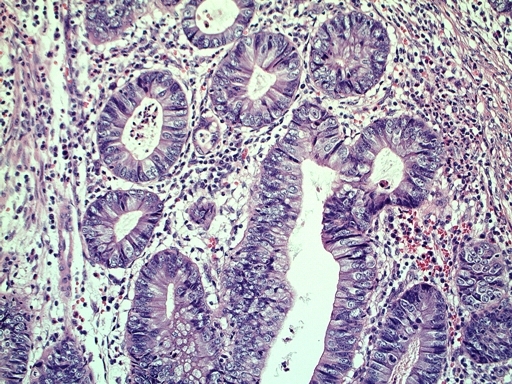
Grading—low-grade malignant polyp (G2).

**Figure 4:**
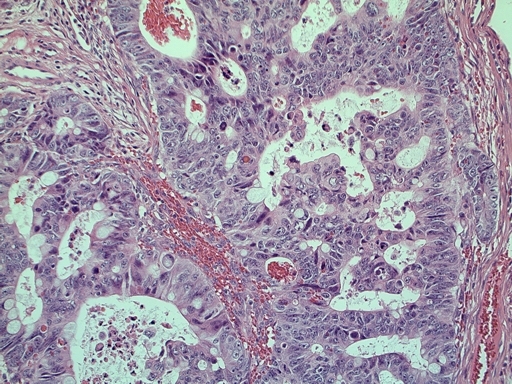
Grading—high-grade malignant polyp (G3).

**Figure 5:**
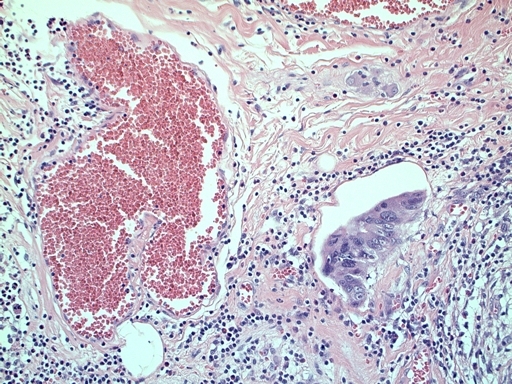

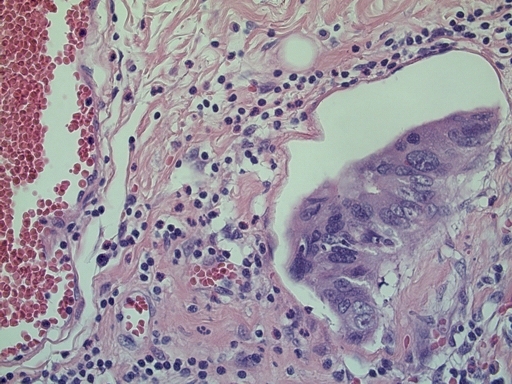

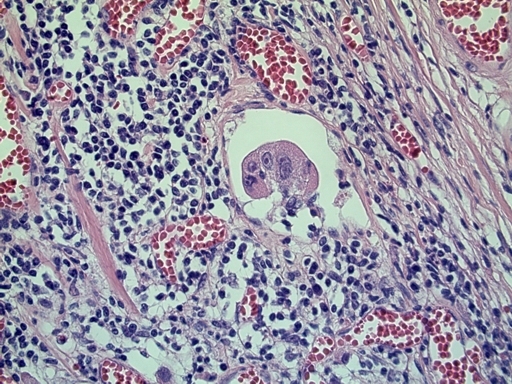
Vascular invasion (a–c).

**Figure 6:**
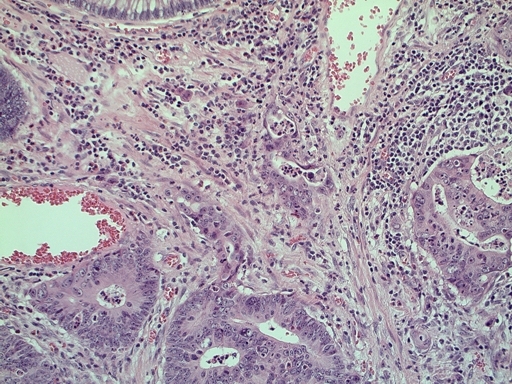

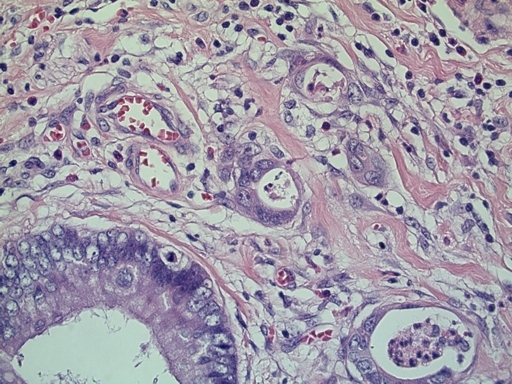
(a) Budding; (b) Low-grade budding.

**Figure 7:**
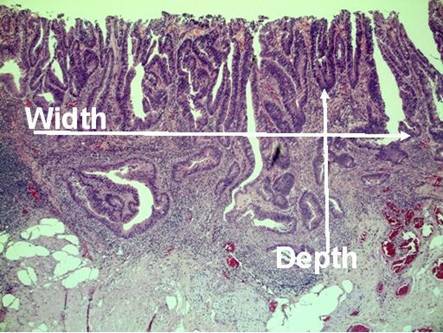
Microstaging—infiltration width and depth.

**Figure 8:**
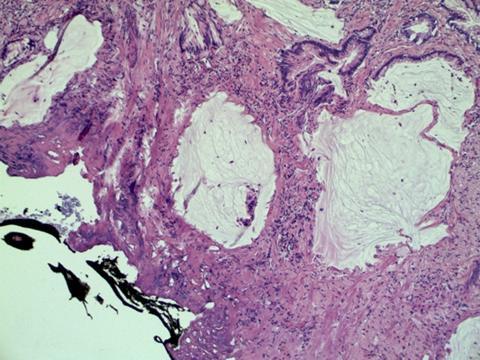
Positive margins.

Figures 1–8 show histopathological characteristics of cancerous polyps at ‘low’ and ‘high risks’ for nodal metastases. Because of the everyday incidence of these ‘early-stage’ carcinomas, the screening programme led to an improvement in both endoscopic and pathological diagnoses, with a better evaluation of these lesions by endoscopists, pathologists and surgeons who have to manage, with the patient, their ‘clinical risk’.

Endoscopists from screening-accredited centres must acquire great expertise in treating lesions detected in FOBT positive subjects. Videos 1–3 show some endoscopic procedures performed for screen-detected lesions by the Division of Endoscopy of the European Institute of Oncology.

As for the already well-established screening programmes for breast and cervical cancers, Italian experts from different areas created the Italian Group for Colorectal Cancer Screening (Gruppo Italiano Screening ColoRettale—GISCoR), which promotes and quality-controls screening programmes all over Italy.

Through the assessment of screen-detected lesions, it will be possible to understand whether screening only offers ‘earlier diagnosis’ or if there are real ‘biological differences’ between screen- and non-screen-detected tumours. Through the establishment of a tissue bank of carefully collected specimens from all the screening centres, any differences can be analysed by clinical and translational studies comparing screen-detected and non-screen-detected cancers.

As Italian screening programmes are still new, the follow-up of identified lesions is still too recent to allow a comparison on survival (either overall or disease free) and mortality (either disease related or non-disease related). Significant data regarding follow-up of both screen-detected and non-screen-detected tumours will be available in a few years’ time.

**Video 1:**
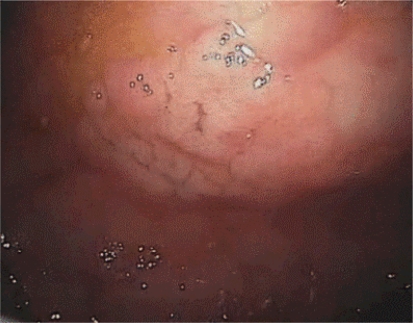
20 mm flat lesion of the caecum. Chromoendoscopy with indigo carmine shows glandular pattern typical for neoplastic adenomatous flat lesions. http://www.ecancermedicalscience.com/view-article.asp?doi=10.3332/ecancer.2009.142

**Video 2:**
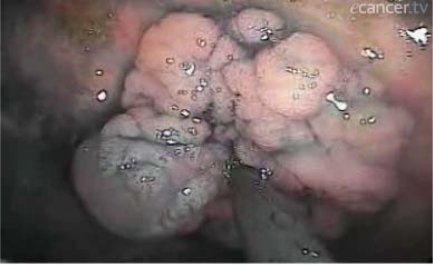
The lesion described above is removed by single-piece mucosectomy after submucosal injection of saline to elevate the flat lesion http://www.ecancermedicalscience.com/view-article.asp?doi=10.3332/ecancer.2009.142

**Video 3:**
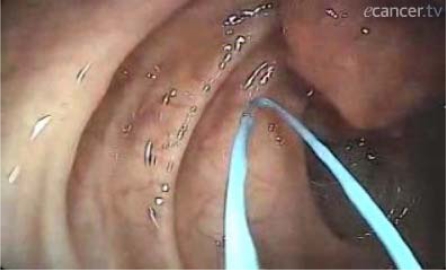
Endoloop-assisted polypectomy to prevent bleeding for a 4-cm peduncolated adenoma with a broad stalk. http://www.ecancermedicalscience.com/view-article.asp?doi=10.3332/ecancer.2009.142

## Figures and Tables

**Table 1:**
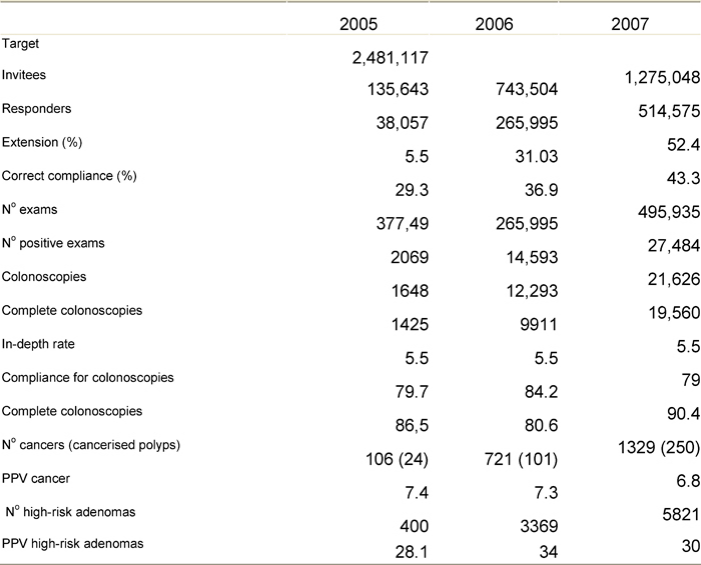
Regional screening programme (Lombardy) 2005–7

**Table 2:**
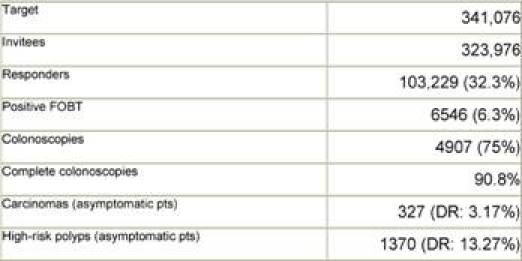
Milan Screening Programme—results after first FOBT round (2006–8)

**Table 3:**
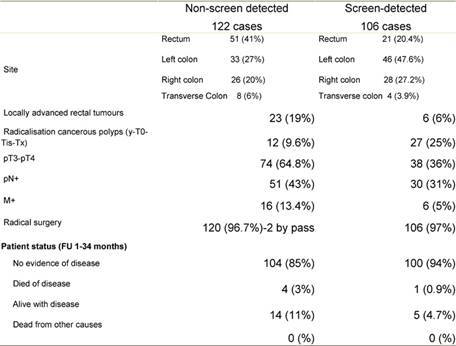
IEO surgical treatment in screen-detected versus non-screen-detected colorectal cancer (from January 2006 to August 2008)
